# Strengthening routine immunization in Papua New Guinea: a cross-sectional provincial assessment of front-line services

**DOI:** 10.1186/s12889-020-8172-4

**Published:** 2020-01-23

**Authors:** Christopher J. Morgan, Olga P. M. Saweri, Nicholas Larme, Elizabeth Peach, Pele Melepia, Lucy Au, Michelle J. L. Scoullar, Mohammad Salim Reza, Lisa M. Vallely, Barbara I. McPake, James G. Beeson

**Affiliations:** 1 Burnet Institute, Melbourne, Australia; 2 Melbourne School of Population and Global Health and Department of Medicine, University of Melbourne, Melbourne, Australia; 3 Papua New Guinea Institute of Medical Research, Goroka, Papua New Guinea; 4 East New Britain Provincial Government, Kokopo, Papua New Guinea; 5 Burnet Institute, Kokopo, Papua New Guinea; 6 World Health Organization, Country Office Port Moresby, Port Moresby, Papua New Guinea; 7 Kirby Institute, University of New South Wales, Sydney, Australia; 8 Nossal Institute of Global Health, University of Melbourne, Melbourne, Australia; 9 Central Clinical School and Department of Microbiology, Monash University, Melbourne, Australia

**Keywords:** Papua New Guinea, Low- and middle-income country, Routine immunization, Campaign, Vaccination, Measles, Polio, Health service delivery

## Abstract

**Background:**

Routine immunization programs face many challenges in settings such as Papua New Guinea with dispersed rural populations, rugged geography and limited resources for transport and health. Low routine coverage contributes to disease outbreaks such as measles and the polio that re-appeared in 2018. We report on an in-depth local assessment that aimed to document immunization service provision so as to review a new national strategy, and consider how routine immunization could be better strengthened.

**Methods:**

In East New Britain Province, over 2016 and 17, we carried out a cross-sectional assessment of 12 rural health facilities, staff and clients. The study was timed to follow implementation of a new national strategy for strengthening routine immunization. We used interview, structured observation, and records review, informed by theory-based evaluation, a World Health Organization quality checklist, and other health services research tools.

**Results:**

We documented strengths and weaknesses across six categories of program performance relevant to national immunization strategy and global standards. We found an immunization service with an operational level of staff, equipment and procedures in place; but one that could reach only half to two thirds of its target population. Stronger routine services require improvement in: understanding of population catchments, tracking the unvaccinated, reach and efficiency of outreach visits, staff knowledge of vaccination at birth and beyond the first year of life, handling of multi-dose vials, and engagement of community members. Many local suggestions to enhance national plans, included more reliable on-demand services, integration of other family health services and increased involvement of men.

**Conclusions:**

The national strategy addresses most local gaps, but implementation and resourcing requires greater commitment. Long-term strengthening requires a major increase in centrally-allocated resources, however there are immediate locally feasible steps within current resources that could boost coverage and quality of routine immunization especially through better population-based local planning, and stronger community engagement. Our results also suggest areas where vaccination campaigns in PNG can contribute to routine immunization services.

## Background

Stronger routine immunization programs are critical to the ambitious Global Vaccine Action Plan (2011–2020), however immunization coverage is not increasing as planned in many difficult settings challenged by expanding childhood cohorts, population displacement by conflict or natural disasters, and limited resources to overcome geographical and infrastructural challenges [1, 2]. With the outbreak of polio in 2018, Papua New Guinea (PNG)’s immunization program faces the question familiar to fragile systems enduring a crisis, that is: how in the wake of a major, rapid emergency response to plan for long-term strengthening of a program that has shown no increase in coverage over the past 15 years. The World Health Organization (WHO) and United Nations Children’s Fund (UNICEF) estimated PNG’s 2017 coverage at 62% for both the third dose of diphtheria-tetanus-pertussis-containing vaccine (DTP3) and the first dose of measles-containing vaccine (MCV1), and at 60% for the third dose of oral polio vaccine [3], contrasting with Western Pacific Regional averages of 97% [4] for these antigens.

Measles, having been suppressed by regular supplementary immunization activities (SIAs) for 8 years, returned to PNG in a major outbreak in 2014, and polio, after a 20 year hiatus, returned in 2018 [5, 6]. In that year, PNG was one of five countries experiencing outbreaks of circulating vaccine-derived poliovirus disease [6, 7], the rare mutated form that results from persistent low coverage with the oral polio vaccine. This has required one of PNG’s largest public health emergency responses, led by the national government with support from Global Polio Eradication Initiative and other development partners [8]. In the short-term, the response entailed repeated SIAs, with longer term plans for program reform, recognizing that it is sustained gaps in routine immunization services that are the primary cause of this form of polio [7].

This paper reports on a cross-sectional health services assessment investigating PNG government efforts to improve routine immunization prior to the polio outbreak. PNG had in 2015 introduced a new strategy to improve routine immunization and reduce reliance on SIAs, termed the Special Integrated Routine EPI Strengthening Program (SIREP) [9, 10]. This sought to improve program performance amidst the major challenges facing PNG’s health services such as a stretched health workforce, financial constraints, and dispersed rural populations with minimal road access [11]. SIREP had four priorities: more efficient local planning based on locations of child populations, intensification of outreach services with realistic scheduling based on local resources, improved local information systems (including child health books), and staff training to support new vaccine introductions (most prominently Inactivated Polio Vaccine (IPV) as well as pneumococcal and rubella vaccines). SIREP also aimed to integrate other primary health care with vaccination, starting with limited curative care, and addressing malnutrition through distribution of age-targeted vitamin A and anti-helminthic doses of albendazole [12]. SIREP attempted to merge the program intensification often seen in emergency campaigns [12] with internationally proven strategies for routine programming, such as those collated by WHO in “Reaching Every District (or Community)” [13] approaches and the Global Routine Immunization Strategies and Practices framework [14]. Our study aimed to investigate the relevance and effectiveness of SIREP in strengthening routine immunization through an assessment of front-line services. Such assessments are important to help guide the improvement of routine systems; an important longer-term complement to emergency responses such as those for polio.

## Methods

### Study concept and objectives

Our study focused on modifiable aspects of front-line services recognizing that, for coverage to increase, strategies to strengthen health system elements must ultimately enable changes in service delivery and uptake. Study objectives were to measure current infrastructure, equipment and supplies for preventive care in infancy; assess what health workers know, think and do in relation to providing postnatal and infancy services; assess health workers’ responses to new strategies; and identify opportunities for health system strengthening and improved quality of care. We devised an evaluation framework that explicitly tested important elements of the SIREP strategy (noted in the Introduction), and that also referenced WHO standards for routine immunization programs [14, 15].

### Study setting and timing

Our study setting was East New Britain province (ENBP), part of a large island in PNG’s north-east, whose population (approximately 393,000) [16] live in small towns and rural villages in highland and coastal topographies. Immunization is usually provided through urban clinics, small rural health centres, scheduled outreach to community sites, and hospital children’s outpatients. ENBP performs relatively better than other PNG provinces on some indicators (such as skilled attendance at childbirth), but immunization is similar to national estimates with 60% of children receiving DTP3 and 48% MCV1 in 2016 [17]. Our research was nested within a long-term multi-partner research initiative in ENBP termed Healthy Mothers Healthy Babies (HMHB) that examines broader issues of women’s and children’s health.

We carried out a cross-sectional assessment of health facilities, staff and clients from November 2016 to January 2017, timed to follow the initial rounds of SIREP training and implementation. Sites surveyed comprised 12 clinics providing immunization services, nine in fixed facilities and three community outreach sites; purposefully chosen as they are all linked to the health institutions providing approximately 80% of maternal and child health care in ENBP [16]. Respondents for interview were chosen to provide a mix of seniority across front-line positions (Table 1).

**Table 1:** Data collection, respondents and tools

*Study subjects*	*Number*	*Details*	*Tools*
Primary health care staff	6	Health Extension Officer or Specialised Nursing Officer	Semi-structured interviews (mix of quantitative and qualitative fields)
	6	Nursing officers	
	6	Community Health Worker (CHW)	
Family members	67	Caregivers of infants being vaccinated, 66 female 1 male	Focus group discussion (10 groups)
Health clinics and their operations	9	Child or family health clinic, static	Audit of infrastructure and equipment against PNG standards as described in national EPI plan [11]. Observation of general clinic procedures (12 sites) Observation using WHO Immunization Session Checklist (11 sessions) Observation of patient flow and staff-patient interactions (15 patients)
	3	Mobile child health clinics (run by staff from the above sites)	

### Data collection tools and ethical considerations

Table 1 summarises the mix of interview, focus group discussion, structured observation and records audit. All tools reflected our evaluation framework by incorporating items testing core elements of the SIREP strategy, PNG’s national immunization plans [11] and WHO standards for program monitoring [15], refined through consultation with national and provincial health managers. We also adapted WHO’s 2015 Immunization Session Checklist [15] into a structured observation tool, devised a local tool to observe patient flow, and used concepts from realist evaluation to deepen interview questions with probes seeking respondents’ views on context and mechanisms of change.

Data collection was carried out by trained local research officers using electronic tablets supplemented by paper-based note-taking. The research team included policy-makers and managers at national and provincial levels; however these investigators were excluded from front-line data collection or initial analyses, to avoid bias. Research officers were trained in quantitative and qualitative data collection, including techniques to minimise social acceptability bias, and to assess and counter perceived power imbalance between themselves and interview subjects. All free-text interview responses were recorded verbatim, focus group discussions were documented by a dedicated note taker, and both were digitally recorded for cross-checking. Interviews were conducted in English and focus group discussions in Tok Pisin; both official languages of PNG.

### Analysis

Quantitative measures from audit, structured observation and interview elements (such as for knowledge) were reported as proportions with no further statistical manipulation, respecting the purposeful sampling in our design. Thematic analysis of qualitative data was carried out by the first author (after translation of focus group discussion data), then validated by three other investigators (including two Tok Pisin speakers). After coding for themes predetermined in our conceptual framework and design, data were re-examined for emergent themes. In 2017, for critique and validation, a detailed data report was provided to all investigators and a summary provided to national and provincial stakeholders; this also allowed early dissemination of policy implications. Additional file 1 is available detailing tables of all themes derived from interview and focus group discussions, and data structure for the observational tool.

## Results

Key findings from all data sources are summarised under six categories (Table 2) from our evaluation framework that combined priorities addressed by the national SIREP strategy [5, 11], WHO program advice [15] and the results of our thematic analysis. We present quantitative and qualitative findings in combination, to show the overall finding against each element of the evaluation framework, in particular the degree to which SIREP objectives were seen in practice. Findings were additionally categorised as either local strengths or areas needing improvement, with decisions on this allocation being made by the research team. A comprehensive table of findings and themes is available as Additional file 1, Tables 1 and 2.

**Table 2:** Key findings, categorised by study themes, encompassing SIREP and WHO program improvement priorities

	*Strengths in local systems*	*Opportunities for improvement in local systems*
Local service planning and delivery *Data sources: interview and audit*	• Recent innovations recognised by most (14/18) staff: new vaccines or planning, with one mention of new quarterly outreach increasing efficiency • Recent in-service training in SIREP reported by many (11/18) staff • Static services available 5 days per week • All clinics tallied vaccinations to report into national health information system • 82% of clinics conducted as planned (annual total of 109 implemented of 133 planned)	• Outreach was limited - minority (2/9) static facilities used outreach with overnight stays for remote areas • Outreach planning process not able to be described by half the staff, less than a quarter planned on population basis, no clinics used population data to estimate outreach supply quantities • Many clinics with few patients: mean 17/clinic, IQR 3–26, maximum = 62 • Estimation of coverage impossible for more than half (10/18) staff due to lack of catchment data, only one clinic displayed coverage • No clinics with lists of children overdue for vaccination, one third of clinics used child registers • Local reasons for clinic cancellation were adverse weather, lack of transport or slow disbursement of funds
Infrastructure and supplies *Data sources: interview, audit and observation*	• Most clinics had road access, with two outreach clinics on walking trails • Water supply in nine (of 12) clinics and electricity in eight (of 9) static sites • Functional injection equipment, safety boxes and weight scales in all clinics • 10 (of 12) clinics with appropriate, functioning cold chain equipment • Supplies of all relevant vaccines (including IPV and PCV) and injection equipment present • No expired or discontinued vaccines found	• Eight (of 12) clinic sites needed renovation (by local standards), 9 (of 12) did not have usable toilets • No clinics with kits for managing severe acute adverse events • Cold storage monitoring inconsistent, no clinics with written temperature records • Half the clinics had clear records of vaccine stock usage, not well reconciled with tallies of patients vaccinated, none able to match supply to population • Recording forms did not clearly account for three new vaccines: IPV, PCV, MR • Lack of important guidance documents: one (of 12) had an immunization manual, and four had child health standard treatment guidelines
Staff knowledge and staff practice during immunization sessions *Data sources: interview and observation*	• More than half of staff could correctly cite recently introduced vaccines (11/18) and handling of lyophilised multi-dose vials (15/18) • Twenty staff (nurses and CHWs) across 12 clinics, vaccinating mean of 17 children per clinic falls below WHO maximums (30 per staff member [15]); • Core interactions (weight, screening and vaccination) done for 14 of 15 observed patients • Observation against WHO session checklist (Fig. 1) shows key elements of safe injection in over 80% sessions	• Less than half of staff could correctly cite immunization schedule (8/18), one national program target (3/18), storage temperature (6/18), interpret vaccine vial monitor display (3/18), or handling of liquid multi-dose vials (2/18) • Some important functions omitted in patient flow observations: educational interactions observed for just two of 15 patients, preventive care for mother and AEFI monitoring observed in none • Waiting times in 15 patient flow observations were significant: mean 51 min arrival to final interaction (IQR 13–90, maximum 210) • Observation against WHO session checklist sessions shows gaps in preparatory checking of vaccines, client communication and AEFI observation, and (for less than 20%) in safe injection
Missed opportunities for vaccination *Data sources: interview, audit and observation*	• Most staff (13/18) stated they would open a multi-dose vial for just one patient	• Due vaccinations had been missed in 3 of 10 Child Health Record books • Reasons for missing vaccination included vaccine out of stock, clinic visit not for purpose of vaccination, or (for birth doses) childbirth in community • Two (of 15) observed patients asked to return another day for vaccination • Thirteen (of 17 respondents) staff stated they would usually ask a sick child to return at another time for vaccination
Integration of other services *Data source: interview and* *observation*	• All staff noted a policy of integrating other care and vaccination • Nine (of 18) staff cited at least one other care (usually childhood illness) regularly integrated • Child illness care available in nine (of 12) clinics • Child illness care accompanied vaccination in seven (of 15) observed patients • Weight measured in all observed patients	• Four (of 18) staff mentioned maternal health service as integration option • Three clinics (of 12) lacked supplies, or service organization, to integrate childhood illness care with vaccination • No observation of catch-up vaccination with children presenting for illness • Three (of 15) patients observed to receive feeding counselling, two to receive vitamin A, 1 to receive albendazole • No observations of maternal preventive care or counselling • Staff reported insufficient numbers as main constraint on integrated care
Community engagement and family viewpoints *Data sources: interview, observation and focus group discussion*	• Three (of 12) clinics provided verbal group health education alongside vaccination sessions • Three (of 18) staff reported support (for example food) provided by local communities • No fees for vaccines reported or observed and families did not report fees as barrier to vaccination	• No reported use of community-based trained lay health workers to help with organization, mobilization or education. • Six (of 12) clinics charged small administrative fees • Many families (6/10 groups) cited travel time, and transport costs, as significant constraints on timely attendance for vaccination • Many families (6/10 groups) sought more mobile clinics, more “on-demand” vaccination, and more reliable clinic timing • Some families (4/10 groups) sought more respectful staff-client interactions • No male family members were observed in attendance, staff cited embarrassment as a constraint on male involvement

Notes: *SIREP* Special Integrated Routine EPI Strengthening Program, *IPV* Inactivated polio virus vaccine, *PCV* Pneumococcal vaccine, *MR* Measles-rubella combination vaccine, *IQR* Interquartile range

### Local service planning, infrastructure, supplies and staffing

Findings demonstrated that services were largely provided as planned from static clinics and some outreach sites (Table 2). Several key aspects of SIREP were not yet implemented; there was limited intensification of outreach, no selective targeting of population concentrations, and minimal systems for tracking and finding under-vaccinated children. Infrastructure and supplies review showed a functional level of infrastructure, equipment and supplies across almost all operating clinics, as judged against standards in the national EPI plan. New vaccines were being deployed in keeping with the SIREP strategy and, reassuringly, no expired or discontinued vaccines (such as trivalent oral polio vaccine) were found. However there were important unmet needs for renovation, equipment renewal, improved temperature monitoring, and availability of guidance documents. A significant gap was the absence of equipment to manage severe adverse events following immunization (AEFI). Overall staffing was within WHO global standards for workload (30 vaccinees per staff member [15]) at current levels of operation, noting a number of clinics with relatively few patients (25% seeing three or fewer).

### Staff knowledge and service delivery practices

**Fig. 1:**
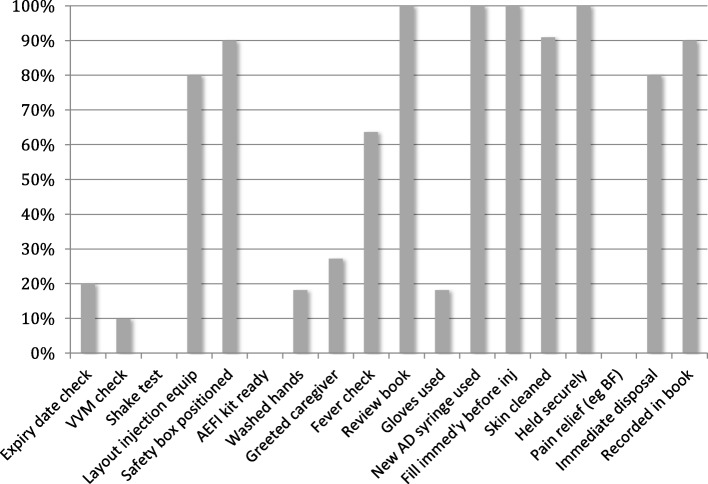
Health worker practices assessed against the WHO Immunization Session Checklist (percentage of provider-client interactions showing the practice). Note: Structured observation of 11 immunization sessions, using the 2015 WHO Immunization Session Checklist [15], which aims to avoid the most common programmatic errors reported by senior immunization managers in a WHO consultation. VVM = vaccine vial monitor, AD = Autodisable, BF = Breast-feeding

Interviews (Table 2, with additional quantitative detail in the Additional file) showed some staff recognising SIREP objectives, many staff understanding the essentials of safe and effective vaccination, and all staff reporting recent refresher training. Important knowledge gaps related to vaccines given at birth or in the second year of life, the links between vaccination and disease control, and proper handling of liquid multi-dose vials (with the risk that usable vials would be unnecessarily discarded). Structured observations tracking staff-patient interactions for 15 patients and applying the WHO Immunization Session Checklist to 11 immunization sessions (Fig. 1) demonstrated that while most staff provided a clean and effective vaccination injection, there were gaps in provision of education, counselling of families, AEFI readiness, and the checking of vaccines for heat or freezing damage. Several missed opportunities for vaccination were noted, as listed in Table 2.

Most staff at interview reported integration of other services with immunization (Table 2) as important, but observation found limited practice. Although national guidelines recommend integrated care for all infants [18], it was observed that: child illness care was provided alongside vaccination for approximately 50% of infants; while most infants were weighed, counseling on feeding or growth was uncommon; vitamin A and albendazole was distributed less often than scheduled; and no instances of integrating care for the mother were seen. Staff reported lack of time and personnel as the most important constraints.

### Community engagement

As listed in Table 2, community engagement activities consisted of group education talks at 25% of clinics, but without use of pictorial, video, participatory, or take-home communication products. Local communities donated in-kind support to some clinics, but there was no structured engagement, nor use of trained community health volunteers. Small fees (generally between USD 0.3–1.0) for attendance, but not for specific vaccines, were seen in one third of clinics, but were not reported as a major barrier to access. Male parents or care-givers were rarely involved in immunization visits. Community members reported lack of support from family members as an occasional barrier to vaccination.

### Locally generated ideas for improvement

All participants (staff in interviews and community members in discussions), when prompted were able to describe local strengths or suggest improvements. Common ideas proposed by staff included more personnel, stronger support for transport needed to do outreach, better community engagement, more active community education, involvement of male parents or care-givers, and offering desirable extra services for both mother and baby. When asked to rank priorities, staff rated reliable vaccine supply and renewal of equipment and infrastructure as more important than staff numbers or staff knowledge. Care-givers sought more frequent, reliable and ‘on-demand’ services, especially outreach, noting travel time and costs as a common barrier to uptake. Mothers, more often than staff, asked for the addition of family planning, promotion of reproductive health, and maternal illness care at immunization clinics.

## Discussion

Our findings depict an immunization service with an operational level of staff, equipment and procedures in place; but one that reaches just half to two thirds of its target population. The service strengths are similar to, or better than, many other sites in PNG [10], and the service deficiencies are similar to those identified for other low- or middle-income settings [1]. These findings point to a range of immediate opportunities to improve coverage and strengthen local service quality. We discuss these below and, in Table 3, synthesise them into ten recommended actions. By using an evaluation framework based on both PNG’s SIREP strategy and WHO standards, it is possible to relate recommendations to what was included in PNG’s SIREP strategy. Six of our ten proposed actions were already embedded in the SIREP strategy (and hence in national immunization plans), but insufficiently resourced or implemented in our study setting. Particular resource gaps lie in resourcing for outreach. Four actions proposed go beyond the current SIREP strategy.

**Table 3:** Suggested local actions to improve routine immunization program coverage or quality, and potential contribution of emergency responses or campaigns, in rural Papua New Guinea

*Local actions already proposed in the PNG government’s SIREP strategy*	*Potential contribution of emergency responses or campaigns*
Local planning based on populations rather than geography	Campaign coordinators help boost local routine planning capacity Mapping child populations and data-sharing
Intensified quarterly outreach focused on higher clinic numbers properly resourced and implemented	Identify new outreach points, especially with population clusters Clarify options and costs for transport
System for tracking unvaccinated children	Mapping child populations and data-sharing
Integrated SIAs with additional vaccines, matching local priorities	Involve district level in planning Local flexibility in an expanded package of campaign services
Supportive supervision linked to refresher training including good communications and AEFIs	Distribute resources to staff Use campaign monitoring to collect staff priorities for capacity development
Trained lay health workers (health volunteers) to track births and children, support outreach clinics and promote uptake at static clinics	Campaign organisation that promotes local involvement Leverage campaign supports to enlist long-term interest and support
*Local actions that go beyond the PNG government’s SIREP strategy*	*Potential contribution of emergency responses or campaigns*
Standardise every opportunity for vaccination, by policy, training and accessibility of vaccine supplies	Not easily addressed by emergency responses or campaigns
Health communication products and programs to educate families on the complete vaccine schedule	Distribute family-oriented communications materials promoting catch-up vaccination
Test models of integrated services, responsive to community preferences	Not easily addressed by emergency responses or campaigns
Review of staff roles and functions to optimise allocations and workload	Minimise incentives that discourage outreach as part of routine programs

Note: *SIA* Supplementary Immunization Activity. *AEFI* Adverse Event Following Immunization

### Options for short-term improvements in existing services

Within current resources, improvements in coverage should be achievable with a fuller implementation of the changes to local service planning envisaged in the SIREP strategy. This recognises that many clinics see few clients (25% with 3 or less), and the fact that most planning is not yet tuned to where most children live. Other persisting gaps in knowledge and capacity that were prime targets of the SIREP strategy include improving catchment population data, increased frequency of service availability, a greater number of outreach points, and quarterly intensification of outreach. Change through improved local planning to reinvigorate outreach has proven successful in settings in Africa and Asia [13] that share similar burdens of disease and health system constraints to PNG. In past programmatic research in PNG, alongside the country’s national coverage survey in 2004 [19] similar potential gains were identified. Reinforcement of SIREP training, already well recognised by front-line staff, appears a helpful starting point, but with a stronger commitment of resources to enable more outreach services. Such changes also meet many of the highest priorities expressed by family members in our study.

Our findings also indicate opportunities for increased community engagement and mobilisation; through increased group and individual counselling in the vaccination encounter and the creation and provision attractive, durable, “take-home” information products, aiming to build community demand for a timely, complete schedule of vaccination. Outreach can benefit from stronger, formalised involvement of local leadership, possibly with the deployment of trained lay health workers; such community resources can also help register and track children needing vaccination. Improved session practices, including ensuring managerial and stock support to enable staff to open multi-dose vials even for one child; and the institution of AEFI equipment and periods of observation (which also allows time for education). This mix of enhanced community engagement plus improved local planning has driven routine immunization improvements in difficult settings in sub-Saharan Africa [20, 21], and vaccination support by trained lay health workers, termed “Village Health Volunteers” in PNG, has been proven in this country in the past [22].

### Interactions between campaigns and the routine program

PNG’s 2018 polio outbreak has necessitated a major emergency response, with national and sub-national campaigns initially for polio vaccination alone and later with other antigens, particularly measles-rubella, similar to previous SIAs in that country [6, 23]. In Table 3, we have suggested where, based on our findings, emergency responses or campaigns could synergise with the proposed actions to strengthen routine immunization. In other settings, the “micro-planning” used in polio and measles campaigns can inform local service planning [13]. Such planning was flagged in PNG’s SIREP strategy but not fully implemented in our study site. Sharing of campaign coordination staff and systems to support local managers could help catalyse change by identifying new outreach sites, rebuilding local clinic registers and catchment descriptions, and setting benchmarks for sustainable, practical transport costs. Campaigns and emergency responses could also work for stronger community engagement, including communications with local leaders and trained health volunteers that advocate for long-term support to the continuing routine program. Other practical support could address the planning and information gaps noted in our study, by distributing Child Health Record Books, staff immunization manuals, and other key knowledge resources needed by the routine program.

There is global evidence, especially for measles/rubella SIAs, that they can boost routine programs [24]; but only if they accommodate the needs of routine immunization in the way they harmonise their planning, invest in suitably generic equipment, share staff and intelligence, use broadly supportive communications, and minimise unsustainable monetary incentives. PNG’s past experience with SIAs suggest they had most success when they maximised district-level control of timing and operations, and of which package of services to integrate [25, 26].

### Longer-term issues

It is clear that changes limited to the front-line are insufficient and central reforms of management, a country-led technical advisory group, procurement and financing, and national re-equipping are also needed and have been repeatedly advised [10, 11, 27]; these were largely beyond the scope of our research. Our findings do illuminate the need for new thinking on the immunization workforce; in interviews staff consistently mentioned lack of personnel as an important constraint on extending outreach or integrating new services, in contrast to our observations that staffing was more than adequate for the clinics actually operating. Expanding services will eventually require an expanded cadre of vaccinators, but prior to that our findings suggest a need to expand immunization responsibilities among existing staff, in pursuit of greater efficiency. This could be coupled with the reestablishment of commitment to the national immunization program goals across all staff levels, as one contribution to a revitalisation of immunization professionalism.

One aspiration of the SIREP strategy, and of global immunization programs [28], is the greater integration of other services with vaccination; seen to a limited degree in our study by the distribution of vitamin A or albendazole. Our community discussion findings reflect a demand for integration that goes beyond this, prioritising relatively complex services such as maternal illness care, or family planning counselling and provision. These require time and skill that seem difficult within current staffing and infrastructure limits that we have mapped, and would seem to need an integration strategy such as service co-location rather than simply adding tasks to current vaccinators. Testing models to address maternal as well as infant needs through routine contacts in the first year after childbirth, appears profitable and important. If integrated service provision prioritises care that families want, this may help build demand for and confidence in immunization services, as well as meeting their felt needs.

### Limitations and strengths

Our sample was restricted to functioning services and families who were willing and able to use those services so our study primarily relates to improving outcomes within existing services. Our data collection took place over the fourth and first quarters of the year, when wet weather events could bias perceptions of access. Despite careful training in unobtrusive observation and non-leading interviewing, there may be some observer effect and/or social acceptability bias affecting the validity of our findings. Single author coding of themes in qualitative analysis increases the risk of bias; our mitigation was to critically reflect on research perspective and carefully cross-check all inferences with local research staff, implementers and policy-makers. Study strengths include the assessment of a representative sample of functioning services, as well as the use of a broad mix of methods and attempt at more detailed interviewing than is the norm in previous service evaluations.

## Conclusions

Our assessment of front-line immunization services in rural PNG found opportunities to boost coverage and quality, even within current resources, especially through better population-based local planning, and stronger community engagement. Many, but not all, were contained in PNG’s recent national strategy (SIREP*)* for routine program strengthening. Our results call for increased resourcing of and commitment to this approach, and also suggest areas where vaccination campaigns in PNG can contribute to routine immunization services.

## Supplementary information

**Additional file 1.** Additional quantitative knowledge data and thematic coding of qualitative data.

## Data Availability

Summary tables of qualitative findings by thematic coding are provided available as supplementary material. Additional datasets used for the current study are available from the corresponding author on reasonable request.

## Abbreviations

AEFI: Adverse events following immunization
DTP3: Diphtheria-tetanus-pertussis-containing vaccine third dose
ENBP: East New Britain Province
EPI: Expanded Programme on Immunization
HMHB: Healthy Mothers Healthy Babies research program
IPV: Inactivated polio vaccine
MCV: Measles-containing vaccine
PNG: Independent State of Papua New Guinea
SIA: Supplementary immunization activity
SIREP: Special Integrated Routine EPI Program
UNICEF: United Nations Children’s Fund
USD: United States dollar
WHO: World Health Organization
