# Supplementary material for: Strengthening routine immunization in Papua New Guinea: a cross-sectional provincial assessment of front-line services
Source: BMC Public Health. 2020 Jan 23;20:100. doi: 10.1186/s12889-020-8172-4 (PMC6979348; doi:10.1186/s12889-020-8172-4)
Supplement: Supplementary file 1 — Additional file 1. Additional quantitative knowledge data and thematic coding of qualitative data. [file 12889_2020_8172_MOESM1_ESM.docx]

# SUPPLEMENTARY MATERIAL

# Morgan C *et al.* Strengthening routine immunization in Papua New Guinea: implications for post-polio renewal from a cross-sectional provincial assessment of front-line services. Correspondence: <chris.morgan@burnet.edu.au>

This document provides one chart with quantitative knowledge responses from health workers, and tables consolidating themes in qualitative data collected through the interviews and discussions described in the main publication. Site-specific information has been removed and respondents assigned numeric codes to ensure anonymity. Additional raw quantitative data is held in secure project archives, available on request.

# SELECTED HEALTH WORKER KNOWLEDGE RESPONSES – COUNT OF RESPONDENTS WHOSE ANSWER ALIGNED WITH NATIONAL POLICY, FOR 17 HEALTH WORKERS

# THEMATIC CODING OF HEALTH WORKER FREE-TEXT RESPONSES

*Thematic coding of free-text responses from 18 health workers involved in managing or providing front-line immunization services, either as their primary function or secondary to their other role/s.*

## SIREP Implications and actions

### SIREP Implications

*Note some responses may conflate SIREP with routine EPI or with other changes to EPI.*

| *Theme* | *Responses* | *HW Codes* |
| --- | --- | --- |
| Planning changes may improve access | 1. It would change the normal immunization routine which is monthly and we don’t get the immunization numbers expected. So the change has made it to go in time for babies immunization scheduled dates 2. Cut down on cost for families to access vaccinations in town. Health workers will better monitor the number of children accessing immunization and plan accordingly 3. Because of SIREP Program and we are conducting immunization daily instead of weekly as traditionally practiced. | 1. 17 2. 7 3. 2 |
| Potential to reduce disease | 1. Less disease with immunization 2. Improving health of babies in community 3. Will reduce workload at the children’s outpatient because immunization protects children from illness 4. It will help stop measles out break | 1. 12 2. 11 3. 8 4. 3 |
| Introduces new vaccines | 1. New vaccine knowledge 2. It would change the plan of schedule and we learn about new vaccines. 3. 1.1 Additional practice to immunization | 1. 18 2. 16 3. 4 |
| Reduces health service costs | 1. SIREP can be scheduled and therefore the Government will spend less 2. Will reduce workload at the children’s outpatient because immunization protects children from illness | 1. 7 2. 8 |
| Potential for integration | 1. SIREP Program will help to integrate the services within the clinic and during the outreach | 1. 9 |
| Potential for less antibiotic resistance | 1. The SIREP Program will help to reduce the use of antibiotic on babies and therefore help to reduce the risk of drug resistance amongst babies | 1. 7 |
| Adds to workload | 1. It is an additional work to me. 2. It needs more time and to program schedule especially for man power 3. It would affect the manpower 4. It’s an added workload to me | 1. 18 2. 14 3. 13 4. 10 |
| Not aware | 1. Was never part of the SIREP so cannot really tell 2. Was never part of the SIREP so cannot really tell 3. Was never part of the SIREP so cannot really tell 4. We only attend to well-baby and opportunistic 5. Was never part of the SIREP | 1. 12 2. 8 3. 5 4. 6 5. 1 |

### What SIREP needs to be effective

| *Theme* | *Responses* | *HW Codes* |
| --- | --- | --- |
| More staff (to match population) | 1. We need more man power 2. Need to improve manpower so we can fully implement all services 3. Increase staff ceiling for this facility because population is increasing 4. Need to have more manpower 5. Need more man power | 1. 18 2. 16 3. 15 4. 13 5. 10 |
| Dedicated preventive care services | 1. Establish well baby clinic and mothers health check-up, so when they bring their children for check-up, they are checked as well *[general OPD]* 2. Someone must be assigned full time to provide vaccination, Well baby clinic needs to be established 3. Have a separate wing in the facility that will be able to look at such programs and have Well baby clinic alone since currently both well babies and sick babies go to one area only to be checked and sent for treatment 4. Improve services on measles | 1. 13 2. 10 3. 9 4. 3 |
| Transport resources | 1. We need constant transport to effectively implement SIREP program. 2. This SIREP Program is worse in some areas that they cannot easily access some villages like in the bainings or Kerevat remote areas or Pomio | 1. 16 2. 9 |
| Vaccine availability | 1. Availability of vaccines. At the moment there is no PCV/IPV and MRV 2. All Vaccines must be available at all times | 1. 7 2. 8 |
| Communication efforts needed | 1. Awareness/toksaves has to be done and notices putout or given out to the ward members and LLG managers 2. Need to improve awareness on when and how to give | 1. 17 2. 11 |
| Community engagement | 1. attendance of mothers bringing their children | 1. 4 |

### SIREP in-service done post training

| *Theme* | *Responses* | *HW Codes* |
| --- | --- | --- |
| In-service passed on to MCH staff | 1. Inservice has been done 2. All staffs under MCH have been in-serviced on SIREP and are now knowledgeable in all new vaccines 3. Yes, nursing officers who attended the training have trained us 4. Yes, staff who attended the training have trained us in the facility 5. Second In-charge of the facility who attended the training shared the skills with staff 6. Yes, SIC attended the training and imparted her skills to all the staff 7. Yes, I did train the staff at my facility 8. Three staffs were trained in the these facility who were then part of the SIREP Program so training was done by the 3 staffs to the other staffs and therefore they disseminated the information to other staffs 9. Yes, SIC conducted in-house training for all staff at ANC 10. 1.3 Yes, SIC did in-house training to all the staff at the facility 11. Yes, I attended the training and did in-house training for all the staff | 1. 17 2. 18 3. 16 4. 15 5. 14 6. 11 7. 10 8. 9 9. 8 10. 4 11. 2 |
| Secondary training not reported | 1. I did not attend the formal training but was involved in the actual implementation of SIREP program 2. Was never part of the SIREP so cannot really tell 3. Was never part of the SIREP so cannot really tell 4. Was never part of the SIREP so cannot really tell 5. We were not given any chance attend RIREP training that is why SIREP is done by MCH staff. 6. There was no training done for us. Nurses were just appointed to be part of the SIREP program and the person who was train will do the immunization herself 7. Was never part of the SIREP | 1. 13 2. 12 3. 8 4. 5 5. 6 6. 3 7. 1 |
| More training needed | 1. But we need in-house training 2. Need constant in-service on immunization and ensure everyone understands new changes in vaccines 3. No proper training done but was just given information on the dosage and age group | 1. 14 2. 11 3. 7 |

## Immunization service coverage and quality

### Most important constraints

| *Theme* | *Responses* | *HW Codes* |
| --- | --- | --- |
| Vaccine shortage | 1. Vaccine shortage and weather is a main problem faced in delivering the immunizations to babies. 2. Shortage of vaccine supply at area medical store 3. Main factor is supply of vaccine stock is not up to date to provide good immunisation 4. Inconsistent supply of vaccines Vaccine supply is out of stock. 5. Only the supply – some vaccines are not in stock at times 6. Vaccine stocks (Limited/not enough) 7. insufficient supply of vaccine from the provincial health | 1. 17 2. 18 3. 16 4. 14 5. 6 6. 3 7. 2 |
| Cold chain infrastructure | 1. We do not have a vaccine fridge and less manpower *[general outpatient]* 2. No vaccine fridge so just using the cold box therefore cannot have all the vaccines needed to support the immunization coverage 3. We do not have a vaccine fridge, we only use cold box to store vaccines at the facility 4. Storage of Equipment especially vaccines-Storage facility for vaccines. All other areas are coming to get vaccines here at this facility because other facilities don’t have a proper storage freezer for vaccines - it’s worse in other facilities when it comes to cold chain 5. Main problem is the facility is using domestic fridge to store vaccines and if main power supply goes out for several hours is not good we need a vaccine fridge. 6. We need a new vaccine fridge instead of domestic | 1. 13 2. 12 3. 11 4. 5 5. 4 6. 2 |
| Transport resources for reaching remote populations | 1. Transport, Ambulance is 13 years old and need proper maintenance 2. Transport is not always available 3. Lack of vehicle to transport community mobile sites and at the moment we use one ambulance 4. Physical environment such as scattered villages, mountains and hills is another barrier 5. Physical environment – poor transport, scattered villages and lack of funds for transport 6. No road access to many villages who are leaving right inside. Our transport cannot go right in and therefore coverage is not good. It’s worse in the bainings and pomio | 1. 14 2. 12 3. 11 4. 10 5. 6 6. 1 |
| Staff shortages | 1. Shortage of staffs or lack of staffs 2. Man power shortage 3. Man power shortage 4. Less number of staff and when plenty mothers attend clinic for immunization, sometimes we don’t provide health education and proceed straight to vaccines 5. Staff shortage so no one to help give immunization or do MCH | 1. 12 2. 11 3. 10 4. 8 5. 3 |
| Staff attitudes | 1. Staff shortage due to sickness, other social or cultural events that pops up 2. Staff punctuality and timing is currently a problem. If staff turn up on time then they will be able to catch mothers on time 3. Staff punctuality is a hindrance to coverage and quality. If staff turn up on time. The coverage will be high and they will be able to provide quality service 4. Attitudes of staff who come to work with personal problems may not perform well or might inject wrong vaccine against what was prescribed | 1. 15 2. 9 3. 7 4. 2 |
| General funding or infrastructure | 1. Lack of funding from government and church structures 2. Funding should be available at all times to carry out programs. Staffs will only attend to clinics/ special programs if there are funds available. These days are so different because people rely mainly on money [incentives] to carry out work which will also help boost their morale to support and give quality services. | 1. 14 2. 9 |
| Difficulties in community engagement | 1. Difficulties in relationship between the community and the staff. Mothers are ignorant. 2. Mother’s Ignorance 3. Mothers commitment during clinic days and therefore don’t turn up at the clinic on the set dates 4. Important factor is mobilisation of parents with their children from one location to another. When mothers are not in one location and a mobile clinic is held at that location then the child is missing her/his vaccine due for that month | 1. 16 2. 9 3. 7 4. 2 |
| Weather, floods, rain | 1. Weather. Continuous rain causes bad road conditions 2. Bad weather prevent mothers from coming for immunization | 1. 17 2. 8 |

### Constituents of a good quality service

| Vaccine stock management | 1. Must have good sufficient supply of stocks of vaccines every month and it must be ordered according to population and expiry dates meaning the vaccines has to be checked of their expiry dates before being sent out the health facilities so we don’t have expired vaccines sitting in our fridges. 2. All vaccines available. Maintain cold chain. Availability of vaccines 3. Maintain cold chain at all times 4. Maintain good cold chain Protect vaccine from sun light Keep at right temperature 5. Vaccines available. If stocks are available then the service flow is constant 6. Keep vaccine in cold chain at all times, ensure thermometers are at right temperature at all time, check VVM all the time 7. Keep potency of fridge by maintaining cold chain at all times 8. Enough stocks of vaccines 9. Maintain temperature of domestic fridge twice a day. Keep a proper record of stock in and out daily. Check VVM regularly | 1. 17 2. 18 3. 16 4. 15 5. 14 6. 11 7. 4 8. 3 9. 2 |
| --- | --- | --- |
| Staffing numbers and capacity | 1. Enough man power 2. Enough staff must be available to implement in the immunization 3. Availability of staffs to provide immunization for all and to provide quality service 4. Staffs with knowledge on vaccines. Staff punctuality. 5. Enough staff to finish work on time, when we give more health it would prevent children from illnesses related to immunizable diseases 6. Availability of staffs to provide immunization for all and to provide quality service 7. Nurse availability, enough supply all the time | 1. 18 2. 14 3. 12 4. 7 5. 8 6. 5 7. 6 |
| Staff skills and training | 1. training staff on new vaccines so they give right vaccine per route and handle vaccines in a proper way 2. Availability of staffs to provide immunization for all and to provide quality service 3. Staffs with knowledge on vaccines | 1. 13 2. 12 3. 5 |
| Effective, supported outreach | 1. vaccines and transport available 2. Clinic patrols would make up a good quality service with separate ambulance for patrol alone. Currently the MCH is using one ambulance so at times it is called off for immunization 3. Good planned program with no cancelations of MCH 4. funds will help to purchase transport to transport vaccine | 1. 14 2. 7 3. 4 4. 1 |
| Dedicated preventive care | 1. Establish well baby clinic and mothers health check-up, so when they bring their children for check-up, they are checked as well *[general OPD]* 2. Someone must be assigned full time to provide vaccination, Well baby clinic needs to be established | 1. 13 2. 10 |
| Community aware and supportive | 1. Mothers must be aware of their next clinic dates 2. Good communication between the staff and the local community 3. Health awareness on review dates for vaccine | 1. 16 2. 14 3. 6 |

### Main limit on quality

*May consolidate this with 2.1 if answers did not distinguish adequately from 2.1*

| Same as 2.3 | 1. As above (10 respondents) | 1. 17, 18, 16, 14, 13, 12, 11, 9, 8, 6 |
| --- | --- | --- |
| Insufficient outreach to community level | 1. It would be good to do overnight patrols. Too much relying on transport and funds making it difficult to cover the catchment population. We also need funds allocated to conduct overnight patrols | 1. 17 |
| Power outages | 1. Prolonged power outage 2. Power supply cut off, frequent power outages for so long can destroy the vaccine | 1. 18 2. 15 |
| Community preferences on provider | 1. Some educated clients do not want CHWs to attend to them. Currently, we have 5 Nursing Officers and 4 CHW people prefer NOs than CHWs | 1. 11 |
| Service planning limits access | 1. Number of day immunisation services is provided. Measles are given on Mondays and Thursdays only - this is worse in other catchment areas | 1. 5 |
| Staff knowledge | 1. Knowledge – most staff are still confused with introduction of new vaccine on when to administer 2. Gaps in staff knowledge. We still have aging staffs who have limited knowledge and skills. Because many aging staffs still follow the old training and skills and takes time to adapt to new changes. Therefore the coverage is poor because of the gaps in staff knowledge | 1. 4 2. 1 |

## Integration of other services with EPI

### Most important additional services

| *Theme* | *Responses* | *HW Codes* |
| --- | --- | --- |
| Family planning | 1. Family Planning is number one on the list of integrating services 2. Family Planning, STI, TB screening and outpatient must be combined with immunization. 3. Family Planning 4. Family Planning to be combined with immunization 5. Family Planning 6. Family Planning because mother can be served as well as well as baby 7. Family Planning 8. Family Planning and health talk 9. Family Planning – both parents must bring their children to clinic so they can be educated at the same time 10. Family Planning because of lack of support from husband, when she brings her child for immunization, she may be served as well at the same time | 1. 17 2. 18 3. 16 4. 15 5. 12 6. 11 7. 10 8. 8 9. 4 10. 2 |
| General health education | 1. Give health talks on different topics (ANCP – BI project very helpful). Health talk on the side effects of immunizations. 2. Health education 3. Including health talk 4. ANC, as inclusion of all relevant health education 5. Family Planning and health talk 6. Health education should be a must with immunization | 1. 17 2. 16 3. 14 4. 9 5. 8 6. 6 |
| STI | 1. STI outpatient must be combined with immunization. | 1. 18 |
| TB | 1. TB screening and outpatient must be combined with immunization. | 1. 18 |
| Nutrition general | 1. Nutrition | 1. 16 |
| Breast feeding and infant nutrition | 1. Advice mother on breastfeeding and nutrition. Explain to mother on weight graph with immunization. | 1. 15 |
| Antenatal care | 1. Combine ANC with immunization 2. Antenatal 3. ANC to be integrated with all other services since the program has all the educational information | 1. 14 2. 12 3. 9 |
| Involving fathers | 1. help both mothers and fathers to realize the importance of health care around mothers, babies and the family as a whole 2. Family Planning – both parents must bring their children to clinic so they can be educated at the same time | 1. 9 2. 4 |
| General illness management | 1. and outpatient must be combined with immunization. 2. Outpatient | 1. 18 2. 12 |
| Every opportunity rationale | 1. Because it’s by chance that they came so we must treat problems as well, so it makes easier for them. | 1. 18 |
| Client convenience rationale | 1. So it makes easier for them. 2. So she does not have to come back | 1. 18 2. 11 |
| Co-located services | 1. Callan services (Disability). Ante-natal should happen. VCCT should happen. Family planning should happen | 1. 7 |

### Constraints on integration

| Staff shortage, staff diverted to other care | 1. Staff shortage is the main problem 2. Staff shortage is the main problem we have 3. Manpower is the main factor that prevents additional services with immunization 4. Staff shortage is 1 main problem that is happening 5. Staff shortage because if one nurse is busy with immunization, then she does not have enough time to do FP 6. There is not enough staff to carry out responsibilities specifically in one section. One staff can be assigned to 2-3 sections making it difficult to provide many services all at once 7. Availability of staffs to conduct ANC and no proper facility in the community to conduct ANC 8. Manpower is the main factor, we need more staff 9. Because of lack of staff vaccinator is pulled out during immunization to help with COPD 10. Main factor is insufficient staff to provide immunization and Family Planning at the same time. | 1. 16 2. 15 3. 14 4. 12 5. 11 6. 9 7. 8 8. 6 9. 2 |
| --- | --- | --- |
| Staff time an issue | 1. Staffs to have extra time with the families/ mothers/ babies to provide the proper help | 1. 17 |
| Staff attitude, motivation | 1. Staffs attitude – When there is money funds available to conduct programs, and then they will be happy to provide a service. If not then they won’t be able to provide it | 1. 9 |
| Infrastructure | 1. Physical settings such as no privacy | 1. 10 |
| Institutional policy | 1. Church run institution does not allow artificial family planning methods 2. Main factor barrier is Catholic run facilities have church policies that does not allow other FP methods to be practiced except ovulation method. | 1. 7 2. 4 |

## Planning and organization of mobile clinics and patrols

### Priorities in planning mobile clinics

| *Theme* | *Responses* | *HW Codes* |
| --- | --- | --- |
| Geography, most remote first | 1. Choose clinic site from further distance first and reverse backwards 2. Probably location/ population. Based on distance. Starts off with furthest location with high population area and then to the closest location with low population area 3. Based on distance. Starts off with furthest location and then to the closest location 4. Location will decide where to go for the mobile clinic. SIC is the one who decided where to go according to her own knowledge 5. Provide equal distribution of service by visiting every clinic site per month to ensure all babies due for vaccine are immunized and “I do not go by population” | 1. 10 2. 9 3. 7 4. 1 5. 2 |
| Population size key factor | 1. We follow MCH as rostered but currently we will go by population of babies under one year as instructed by provincial supervisors. 2. Probably location/ population. Based on distance. Starts off with furthest location with high population area and then to the closest location with low population area 3. Go by population and how often to conduct clinic depends on the number of children in one clinic site | 1. 14 2. 9 3. 4 |
| Transport resources to do outreach | 1. Availability of transport to do MCH clinics. It would be good to do overnight patrols. Too much relying on transport and funds making it difficult to cover the catchment population. We also need funds allocated to conduct overnight patrols | 1. 17 |
| Staff movement planning | 1. Decided according to the number of clinics points and if one has 2 clinic points then we decide how to split the staffs | 1. 17 |
| Follow what SIC or OIC says | 1. Sister in-charge of MCH does the mobile schedule and we only follow 2. OIC does the schedule and we only follow 3. OIC does the planning 4. SIC is the one who decided where to go according to her own knowledge. SIC makes the decision on where to go to do the mobile clinic 5. OIC does the schedule so I only carry out the orders 6. I only follow monthly schedule done by SIC 7. SIC is the one who decided where to go according to her own knowledge | 1. 18 2. 16 3. 15 4. 12 5. 11 6. 8 7. 5 |

### Determinants of cancellation

| Weather, including floods/rain | 1. Weather is the main reason for cancellation and SIC makes the decisions and if SIC not around then the OIC makes the call. 2. Bad weather and the OIC has to confirm before we cancel the program 3. Mainly weather since some locations don’t have shelters that can be used during the clinic that the team cannot do MCH during a bad weather | 1. 17 2. 10 3. 7 |
| --- | --- | --- |
| Lack of transportation | 1. No transport also main reason 2. SIC cancel the mobile clinic depending on the situation e.g. no transport available 3. I make the decision to cancel mobile clinic due to no vaccines and ambulance in poor condition or other unexpected events that pops up | 1. 17 2. 18 3. 14 |
| Staff shortage | 1. If no staffs then clinics will be cancelled because only one staff is currently doing MCH and the OIC makes the decision on cancelling the MCH 2. When to stop mobile clinic depends on number of staff available and end of year’s program | 1. 9 2. 2 |
| Vaccine shortage | 1. I make the decision to cancel mobile clinic due to no vaccines and ambulance in poor condition or other unexpected events that pops up | 1. 14 |
| Never cancel | 1. Never cancel any mobile clinic because communities are already informed | 1. 4 |
| Follow what SIC or OIC says | 1. Again it’s the OIC who cancel the mobile clinic 2. Again it’s the OIC who cancel the plan 3. SIC makes the decision 4. SIC makes the decision the cancel the mobile clinic 5. I follow the SIC’s instruction and can’t say much on this 6. SIC makes the decision 7. SIC makes the decision on | 1. 16 2. 15 3. 12 4. 11 5. 8 6. 5 7. 1 |

## Postnatal care

### Most important PNC services

| *Theme* | *Responses* | *HW Codes* |
| --- | --- | --- |
| Personal hygiene | 1. Educate on personal hygiene for mothers after delivery within first six weeks 2. Advice on personal hygiene, treat those who are sick and provide nutritional advice 3. Educate on personal hygiene for mothers 4. Personal hygiene for both mother and baby 5. Personal hygiene | 1. 17 2. 16 3. 12 4. 10 5. 1 |
| Family planning | 1. Education on family planning – Different methods and ovulation within first six weeks 2. advice on family planning 3. Education on family planning – Different methods and ovulation 4. Family Planning. If there is family planning. Mothers will be able to care properly for herself and her baby and therefore prevent neo-natal sepsis and puerperal sepsis 5. Family Planning | 1. 17 2. 15 3. 12 4. 7 5. 1 |
| Routine maternal check | 1. Before mothers discharge, they must have their peri, HB level and fundal height checked 2. observe mothers for any bleeding, lactation, bowl open or not and check for any abnormalities 3. Check mothers fundal height and assess if bleeding a lot or not for 24 -48 hours | 1. 13 2. 8 3. 2 |
| Preventive care mother | 1. Always give iron supplements to mothers because of blood loss during delivery | 1. 4 |
| Maternal illness management | 1. advice to come back if sick 2. Advice on personal hygiene, treat those who are sick and provide nutritional advice 3. check mother for bleeding 4. Postnatal care for both mother and baby, because mother may develop puerperal sepsis or postpartum haemorrhage or breast feeding problem 5. Treatment of infections | 1. 17 2. 16 3. 15 4. 14 5. 1 |
| Nutrition advice (? mostly for baby) | 1. Nutritional advise within six weeks 2. Advice on personal hygiene, treat those who are sick and provide nutritional advice 3. Nutritional advise Check weight graphs on baby’s book to see if baby is growing 4. Breast feeding advice and monitoring 5. Baby must be breast feed as soon as possible | 1. 17 2. 16 3. 12 4. 1 5. 2 |
| Newborn illness management | 1. And the baby may develop neonatal infection or bleeding from cord or breast feeding problem. 2. Treatment of infections | 1. 14 2. 1 |
| Newborn vaccination | 1. Check baby if immunization is given before discharge 2. Immunization must be given straight after birth to prevent from infection | 1. 13 2. 2 |
| Newborn check congenital | 1. Check baby for abnormalities | 1. 15 |
| General health education | 1. Don’t provide PNC but if does the main program that would be good to come in is Education and ANCP program is a preferred program. Because this program will cover almost everything a family should know about on woman’s health and baby’s health 2. Mothers should be well educated on importance on care of baby and herself | 1. 9 2. 6 |
| Outreach visits | 1. Follow up – there should be follow ups for mother and baby for 6 weeks after delivery | 1. 18 |
| Early detection | 1. That’s where early detection of illness from mother and baby | 1. 18 |
| Assumed must be inpatient | 1. Not relevant as we are outpatient 2. We have no inpatient it’s only a day clinic 3. PNC is not included in our duties, its located to Postnatal ward | 1. 12 2. 10 3. 8 |

### Constraints on PNC

| *Theme* | *Responses* | *HW Codes* |
| --- | --- | --- |
| Staff shortage and attitudes | 1. There should be enough staff and transport available to do follow ups. 2. Workload per staff is high therefore we do not have enough time for other vital services 3. staff shortage or when staffs are busy, they do not attend to PNC. For instance, if one nurse is busy with immunization then she does not have time to do peri-care or cord toilet or even give health education 4. Less man power 5. manpower is the main factor that limit us from providing PNC 6. Staffing is a problem and all settings are facing this problem 7. Lack of man power for staff to have enough time for one to provide one to one nursing care. 8. When nurses are busy during this shift may not attend to mothers and her child | 1. 18 2. 16 3. 15 4. 14 5. 13 6. 12 7. 4 8. 2 |
| Facility infrastructure | 1. Setting in the facility e.g. there is no proper bathing place for babies 2. No facility to provide PNC | 1. 14 2. 9 |
| Staff attitudes | 1. staffs punctuality. Staffs performance | 1. 9 |
| Distance to attend HF | 1. Distance of the health facility. Many of the populations live right inside [remote] and transport for them is a difficult thing so they have to walk to get to the health facility | 1. 17 |
| Transport for outreach | 1. There should be enough staff and transport available to do follow ups. | 1. 18 |
| Institutional policy | 1. Catholic Church run facilities do not allow artificial methods of family planning. So cannot provide Family Planning methods to mothers who need it. | 1. 7 |
| Language | 1. Health Education done in Tok Pisin is not well understood and should be done in local language (Kuanua) | 1. 6 |

## Significant changes in immunization

### Significant changes (6.1, 6.2)

| *Theme* | *Responses* | *HW Codes* |
| --- | --- | --- |
| Less disease | 1. The change see is the added vaccination which is PCV13. Since PC was introduce now les number of babies admitted to our wads or seen at the outpatient with PNA. Before when PCV was not yet introduced, a lot of babies were seen with PNA. 2. No more measles cases seen due to Measles Rubella Vaccine 3. Plenty babies are not infected with measles because of Measles Rubella Vaccine 4. new vaccines have minimized the occurrence of diseases that are caused by pneumococcus | 1. 17 2. 11 3. 8 4. 4 |
| New vaccines | 1. The change seen is the added vaccination which is PCV13. 2. more vaccines are available now a days compared to the past. 3. Only one change that was added onto the immunization program is the SIREP Program which introduced the 3 new vaccines MRV/PCV/IPV | 1. 17 2. 18 3. 9 |
| Improved supply of vaccines | 1. more vaccines are available now a days compared to the past. 2. Number of vaccines/immunizations is good and improves coverage 3. Because of SIREP program and we had enough vaccines and now the coverage is picking up. Previously there were not enough vaccines compared to the present 4. Number of vaccines given per month has improved | 1. 18 2. 16 3. 14 4. 2 |
| Changed service organization, including mobile clinics | 1. The SIREP program has change the way mobile clinics are conducted. Before mobile clinics were done monthly and the numbers were not so good so after the change of the schedule to quarterly, we have a good number of babies seen on visits. 2. Increased coverage from SIREP 3. Biggest change is both static and mobile clinic used to provide immunization once a week and now vaccines are given daily | 1. 17 2. 18 3. 2 |
| New preventive care | 1. Don’t have regular well baby clinic so started MCH which is done every day every month. 2. a new intervention was started which is called MEDICAL WELLNESS CHECK. This clinic checks all staffs within the clinic every month for Cholesterol including other checks that should be done for individuals like BP/Urine/Bloods for cholesterol and weight height against the BMI. Other companies are also accessing the Wellness Checks 3. Previously we do opportunists immunization but now because of well-baby clinic and immunization coverage is improving. Now we have nurse vaccinator where we had none before. | 1. 12 2. 9 3. 6 |
| No significant changes | 1. No changes so far for well-baby clinic or immunization since started work 2 years ago. 2. Cannot tell really but from the health secretary report that was given back to the MCH/well baby clinic the coverage currently is 50% only 3. Haven’t seen any changes. | 1. 9 2. 7 3. 5 |

### Reasons why it is seen as significant (6.3)

| Coverage benefits from mobile clinics | 1. More coverage from SIREP 2. Number of vaccines/immunizations is good and improves coverage 3. Mobile clinic is important because we are visiting mothers and babies at home | 1. 18 2. 16 3. 12 |
| --- | --- | --- |
| Outreach opportunity to track population | 1. Child health registration should be kept up to date when doing mobile clinic so to identify accurately on the coverage and provision of immunization | 1. 12 |
| Less disease | 1. helped babies from all the villages and has improved the immunization coverage. As a result no more whooping cough cases seen in the facility 2. Not many babies are sick related to vaccines preventable diseases | 1. 15 2. 8 |
| Provincial targets | 1. It is because we want to reach the provincial target | 1. 2 |

### Good practices from the past

*May combine with strengths questions in Section 7*

| Staff/client ratio was better in past | 1. Before numbers of staff employed were sufficient to implement, now the population is increasing and the rotation of staff per patient is high. Therefore, there is a need to recruit more staff to match the increasing population 2. Manpower in the past was ok but now the population is increasing compared to staff ceiling. Man power in the past years was good to provide all services but now, it is hard 3. Need more man power to effectively implement SIREP | 1. 18 2. 14 3. 11 |
| --- | --- | --- |
| Transport resources used to be better | 1. Transport for mobile services was available | 1. 16 |
| Health talks prior to immunization | 1. Provide health talks to all contact points to mothers before commencing immunization services | 1. 15 |
| Infrastructure in past | 1. if only we have a vaccine fridge in place as we used to do in the past before the second volcano eruption in 2008. | 1. 3 |

## Strengths and improvements

### Strengths at present (7.1, 7.2)

| *Theme* | *Responses* | *HW Codes* |
| --- | --- | --- |
| Commitment to mobile clinics | 1. Mix of static and mobile clinics in order to catch all babies 2. Every day mobile clinic is our strength. Some clinics don’t do mobile clinic that’s why the immunization coverage is low. Our mobile clinic is unique because we do mobile clinic everyday every month 3. Planned mobile clinics are never cancelled, e.g. if there is no ambulance available, we hire PMV bus 4. Transportation for MCH. When the ambulance is not available they hire private cars to transport them to their clinic sites. | 1. 17 2. 12 3. 4 4. 1 |
| Community engagement, multiple channels, respectful care | 1. Health awareness is very important and must be reinforced 2. Engage with community counsellors to communicate with mothers from their wards to bring their babies for immunization and community engagement 3. Community awareness including churches and LLGs on immunization toksave. Create good communication with clients and feel comfortable instead of yelling and screaming at them | 1. 18 2. 15 3. 11 |
| Increased staff knowledge | 1. All staffs under MCH have been in-serviced on SIREP and are now knowledgeable in all new vaccines 2. In –house training, case review and staff participating in the meeting | 1. 18 2. 14 |
| Staff capacity | 1. We have specialist nurses e.g. 2 midwives and 2 HEOs who makes delivery and postnatal care easier. Also most deliveries are done at Kerevat because of midwifes. 2. Currently have enough staffs in the clinic with 1 NO and a NO midwife = 2 Nos and 5 CHWs. Therefore makes it easier for them to have 2 group doing clinics at different sites | 1. 16 2. 7 |
| Advice on immunization at delivery | 1. babies delivered in the clinics so we can be able to map out a good immunization schedule for our clinic points. | 1. 17 |
| Data and records for immunization planning | 1. Supervised delivery to help to have record of all the babies delivered in the clinics 2. Own method for recording children due for vaccination and tracking them from 1 mo to 12 mo. But though they know they have missing children due for vaccines and follow up, they don’t do direct home visits | 1. 17 2. 7 |
| Dedicated preventive service | 1. We have a well-baby clinic and a full time nurse which makes immunization easier for babies | 1. 6 |
| New infrastructure | 1. We have a new facility unlike the past. The new place or settings is convenient to screen babies and vaccinate them within the same settings | 1. 16 |
| Patient flow | 1. Have triage system to identify very sick children quickly 2. TRIAGE – helps me to identify children who are sick, and those who need immunization so other facilities could do the same | 1. 13 2. 6 |
| Cannot think of any strengths | 1. Cannot think of any strength | 1. 9 |

### Suggestions (7.3, 7.4)

| *Theme* | *Responses* | *HW Codes* |
| --- | --- | --- |
| Community health knowledge and engagement promotion | 1. Advice mothers on the importance of supervised delivery 2. Health awareness is very important 3. Both father and mother must be well educated on how to care for baby, importance of immunization, attend regular monthly clinic for health check. Regular health talks on Radio and through churches 4. Give plenty health awareness on various topics on immunization, malaria, Diarrhoea, personal hygiene and TB. I must make it a priority to give health talk every day then others can adapt my way. At the village, my house must be a role model so others can follow. 5. Educate mothers to bring their children all the time during mobile clinics. How we communicate with mothers and the importance of HE and ensure she understands then she will implement what she was told. 6. Provide multiple ways of educational materials such as video, drama, cooking demonstration to family as preventive services. Multiple educations improve peoples’ knowledge on implementation of health talk at home. | 1. 16 2. 18 3. 13 4. 11 5. 8 6. 2 |
| Involving men | 1. Both father and mother must be well educated on how to care for baby, importance of immunization, attend regular monthly clinic for health check 2. Encourage fathers to bring baby and mother to clinic. Set up a well-baby clinic and Men’s Clinic 3. Fathers must accompany mothers and baby to clinic. Sometimes baby miss immunization because mothers might be illiterate so fathers will assist mother for next visit date 4. Support from both parents | 1. 13 2. 10 3. 6 4. 2 |
| Staff numbers and capacity | 1. All facilities should have 3 midwives to help with ANC and Postnatal services. 2. Increase the manpower because population is also increasing 3. Have enough staffing in all section 4. With good staffing currently if one staff is sick they can easily pull out a staff from the other group so they can still do clinics on that day 5. Need more man power including student nurses | 1. 16 2. 10 3. 9 4. 7 5. 4 |
| Improve peripheral services to avoid bypassing | 1. Other facilities must work efficiently so that their population under their catchment area must not by pass their facility to come to us for service. It makes us over worked. 2. There has been mothers and babies from all other catchment areas are coming to our facility to access vaccines 3. Also mothers should be encouraged to go to respective mobile sites instead of pouring into large facilities. Because our coverage rate is high and quickly run out of supply. | 1. 18 2. 5 3. 6 |
| Improve infrastructure | 1. Need a bigger fridge to store enough vaccines to cater for growing population 2. Vaccine fridge at outpatients 3. Domestic fridge should be replaced with vaccine fridge | 1. 14 2. 13 3. 4 |
| Use central coordination and planning channels | 1. The OIC will express this issue during OIC meeting with the provincial leaders how this issue can be solved. It needs to be documented and presented during provincial OIC meetings 2. Write a letter to District Health Manager and Provincial health leaders highlighting the facts and figures about the problems 3. If OIC/SICs have strong leadership roles, they will be able to lead properly in everything in a clinic 4. bring it up to the District and Provincial levels to ensure it takes place because men’s illness is increasing and population as well. 5. We want to reach the provincial target | 1. 18 2. 14 3. 12 4. 10 5. 2 |
| Dedicated preventive care | 1. Establish well baby clinic and mothers health check-up, so when they bring their children for check-up, they are checked as well *[general OPD]* 2. Someone must be assigned full time to provide vaccination, Well baby clinic needs to be established. Set up a well-baby clinic and Men’s Clinic 3. Have a separate wing built specifically for Well Baby clinic since at the moment all babies well and sick go through the children’s out patient 4. Build separate wing for a well-baby clinic and mother and family to get all available service. This makes it easier for mums, babies and families | 1. 13 2. 10 3. 9 4. 1 |
| Integrated services | 1. For effective mobile service, other immunization services combine with Family Planning for mother as well at the static clinic 2. Opportunistic vaccination. Albendazole deworms a child and he grows healthy so make it a rule for children to be given Albendazole at contact points 3. If a midwife could take lead in follow up of mother and child might work with additional staff say 1 or 2 | 1. 10 2. 8 3. 4 |
| Regularity of outreach | 1. We want monthly mobile clinic to reschedule because it makes easier for mothers and babies. 2. Own system for tracking children from 1 – 12 mo, to trace late vaccines | 1. 15 2. 7 |
| Overnight and foot patrols | 1. Should do foot patrols and overnight patrols to catch mothers and babies in the remote areas. Foot patrol and overnight patrols should happen to help cover the immunization to have an increase in the immunization coverage | 1. 17 |
| Vaccine stock management | 1. Order vaccines according to population catchment | 1. 17 |
| Better central vaccine management | 1. Area Medical Store needs to reschedule its supply to provide vaccines to facilities. | 1. 14 |

# FOCUS GROUP DISCUSSIONS WITH CLIENTS

*Thematic coding of free-text responses from focus group discussions with 67 care-givers (one male) attending immunization clinics, 7 at fixed facility sites, 3 at outreach sites.*

## Changes observed in immunization services

This was not an easy question to discuss and some answers were unclear.

| *Theme* | *Responses* | *Sites* |
| --- | --- | --- |
| Improved access | 1. Many mothers are bringing their children to mobile clinic because it makes easier for mothers; mobile clinic is depending on facility’s schedule; mobile Services makes possible for many children to be immunized; well baby clinic at the village has provided accessibility to immunization services at the village and therefore has prevented a lot of babies from getting sick 2. Previous Immunization schedule was three times a day and now its daily, which is good because if I’m busy on Tuesday then can make it on Wednesday; change in the number of days for immunization, before immunization happens only on Wednesdays 3. There is a change in the way immunization is given this days | 1. RM, VS, PM, VS 2. BS, PS 3. VS |
| Staff factors | 1. Approach of staff is good and babies are assessed well | 1. PM |
| No changes noted in immunization | 1. No changes seen in the immunization, we do not see any changes; no changes seen in the immunization, we do not see any changes in the way nurses conduct mobile clinics and their attitude of giving immunizations; no changes seen in immunization; | 1. KS, NS, WS, |
| Changes reported beyond immunization | 1. Fewer babies getting sick 2. Implant is the only change we’ve seen that is a Family Planning Method 3. Malaria program (CBD) has improved the health status of people and people are not spending any money to access the health facility because of testing and treatment availability at the village 4. Cooking oil has been introduce to LBW babies to help mothers to help increase fat intake to help increase the baby’s weight | 1. VS, PS 2. WS 3. VS 4. VS |

## Immunization service – access barriers and suggestions

| *Theme* | *Responses* | *Sites* |
| --- | --- | --- |
| Service planning | 1. We want regular mobile clinics at the village / community; nurses do not follow schedule for mobile clinic; nurses don’t turn up as schedule; we want health workers to conduct regular monthly mobile clinic at each mobile site; we want regular monthly clinic visits 2. Only available one day – should be more; So we suggested immunization must be conducted twice in the facility instead of once a week 3. awareness must be in advance so communities knows when the clinics will be held, no awareness to community that vaccines are out of stock 4. Mothers waited for several hours at the facility to be served by health workers; waiting for 3-4 hours before my child is vaccinated 5. suggest if Aid Post could be provided for immunization and other outpatients 6. Community leaders could take lead in organizing mothers at community level | 1. KS, NS, RM, RS, PM 2. PS, NS 3. KS, BS 4. KS, NS 5. RM 6. PM |
| Transport issues | 1. Not enough money for transport fare; no money to pay for transport fee; pay for bus fares; no money to pay for transport fee; bus fare – plenty kids some are left at home due to cost of transport fee 2. No enough transport going to the community; no bus; some places difficult with transport; no transport/bus; 3. When the weather is not so good then we cannot go the health centre and many mothers are leaving on the other side of the river or are right inside and when the weather is bad, we cannot go to the health centre | 1. KS, RM, WS, RS, VS, PM 2. KS, RM, WS, RS, VS 3. WS |
| Staff factors | 1. Nurses scolding clients for missed vaccines, attitude of staff is not good; attitude of staff is one of the main factor to well-baby clinic; staffs attitude towards mothers and babies has been a problem and therefore causes mothers from accessing the services easily at any time 2. Staff must arrive on time for duty because they are paid fortnightly; nurses must arrive on time for duty 3. Nurses strike, no staff | 1. RS, BS, PM, VM 2. KS, NS 3. RM, WS |
| Supplies | 1. Vaccine supplies run out and I spent money on bus fare and it’s not good; My distance is far and when I come for vaccine found out that no supply so I am sent away; No stock of supply; Nil stock of vaccines. We walk from far and arrive at the clinic only to be told there are no vaccines available. So we go back and it takes us time to return to the clinic because we are living far away | 1. NS, RM, WS, VS, BS |
| Contra-indications | 1. Babies not vaccinated (turned away) when sick; when babies are sick, nurses do not give immunization. They are sent away and advice to come back the next day | 1. PS, PM |
| Community factors | 1. Customs/ other activities in the community during vaccine days; other commitments and therefore don’t attend clinics for vaccinations 2. Pay for clothes to dress baby for health centre 3. At times, ignorance because of laziness 4. all depend again on the mother; no support from husband. They rely mainly on mothers to do all the visits or carry the baby to the clinic 5. Plenty kids and cannot take all of them to the clinic | 1. PM, VM 2. WS 3. VM 4. VM 5. VM |

## Integration – preferences on what to add to immunization services

| *Theme* | *Responses* | *Sites* |
| --- | --- | --- |
| Family planning | 1. We want family planning to be combined with immunization; integrate family planning with immunization; family planning should be integrated; family Planning to be combined with immunization; FP; FP; FP | 1. KS, RM, WS, RS, VS, BS, VM, |
| Women’s health | 1. Combine immunization with mothers general health check; combine obstetrics and gynaecology; general out patient should be done too to check blood and other illnesses; clinic must be held for both mum and children; we want mother’s health to be assessed as well including nutrition and personal hygiene; good to have health care for mothers and babies together 2. Nutritional advice for mothers so they have more information and are to eat from the 3 balance meals a day | 1. NS, WS, VS, PM, VM, 2. VM |
| Women’s illness care | 1. Combine TB, STI 2. mothers will be checked for other illnesses | 1. NS 2. PS |
| Child care | 1. Know what to do when it comes to sickness 2. Breast feeding advice for mothers so they are able to be able to feed their babies better 3. Check baby’s health and to check if the baby has other diseases | 1. VM 2. VM 3. PS |
| Other priorities | 1. Education should be provided to both mothers and fathers so they are able to make better family decisions | 1. VM |
| Reasons why integration favoured | 1. Because of bus fare and lack of transport; because it will help save cost instead of coming back the next day or a different day; to minimise bus fare, as for the working mothers, I want to report to my manager with one medical certificate instead of two. “I can kill two birds with one stone, which is good”; because I don’t want to come back the other day for FP; due to cost of transport fees especially for parents with many kids 2. some mothers are shy to ask for other services like family planning so when they integrate, it will help such mothers 3. because it makes easier for us mothers; because mothers health important 4. Mothers and fathers will be educated so they are able to know what to do when it comes to sickness or immunization | 1. KS, WS, RS, BS, PM 2. WS 3. NS, PM 4. VM |

## Postnatal care – what to check, when and where

| *Theme* | *Responses* | *Sites* |
| --- | --- | --- |
| Preventive care | 1. General Health check for both mother and baby; check if baby is ok generally; proper neonatal care, check for abnormalities or injuries during birth; check new born babies and check general health for babies 2. Family planning; FP services and counselling; 3. Check for reproductive system of mother; reproductive system; mothers health and reproductive system 4. Check our bloods so that we are aware of our blood level after birth 5. Check if mother is breast feeding; breast feeding advice; mother if she is still breast feeding and baby is still breast feeding and check if mother is ok with the breast because sometimes mothers face problems with the breast 6. Check baby if passing waste 7. Nutritional advice for family; nutritional diet for both mothers and babies 8. Personal hygiene | 1. NS, WS, BS, VM 2. RS, BS 3. RM, BS, PM 4. WS 5. WS, VS, VM 6. WS 7. PM, VM 8. PS |
| Pre-discharge check | 1. We want mothers to be thoroughly examined before discharge, examine the baby properly before discharge; health workers to check both mother and baby well before discharge; health workers to check babies properly before sent home, health workers to check mother health well before discharge; 2. All babies must be vaccinated before discharge | 1. KS , RM, RS, 2. NS |
| Post-partum complications mother | 1. Many mums feel abdominal pains when not cleaned properly and some mothers still bleed; check mothers on personal hygiene and what to use when having menstrual period or when still bleeding 2. Malaria; malaria and TB; malaria and AIDS | 1. WS, VM 2. VS, PM, PS |
| Home visit | 1. Want health workers to do follow ups to the village; nurse should check mum while at home; check up to occur at home | 1. RM, WS, PM |
| Other issues | 1. Nurses are too harsh on us during labour and we want them to understand us and treat us with respect. 2. Give pain killers to mothers 3. Health worker must use simple and local language on FP especially TL so mothers understand the consequences well before they can sign the consent. If they use Kuanua (local language) it would be good | 1. KS 2. NS 3. RS |
